# ‘Cubs of Wall Street’: Cocaine Use in Top‐Boy Culture

**DOI:** 10.1111/1468-4446.13212

**Published:** 2025-04-03

**Authors:** Rikke Tokle, Willy Pedersen

**Affiliations:** ^1^ NOVA—Norwegian Social Research Oslo Metropolitan University Oslo Norway; ^2^ Department of Sociology and Human Geography University of Oslo Oslo Norway

**Keywords:** cocaine, ease, gender, high school culture, masculinity, social class

## Abstract

Although cocaine use is rising among youth in many countries, little is known about the social context and its influence on this new pattern of use. Drawing on a theoretical framework of class, gender, and peer‐status dynamics and extensive data from personal interviews, we investigate how cocaine use is culturally situated and socially organised in certain Norwegian high school cultures. The focal sample consists of study participants who stated that they had used cocaine. They totalled 32 persons, of whom 28 were boys. We identify four key cultural characteristics linked to cocaine use: (i) *affluence*: users often had backgrounds rich in economic capital; (ii) a *party‐centred culture*: cocaine was introduced in contexts with excessive partying and binge drinking; (iii) *top‐level networks*: cocaine use was linked to exclusive social networks, based in Norwegian high school graduation celebrations; and (iv) *masculinity*: boys used more cocaine than girls, to boost their energy and self‐confidence. We conclude that the key driver of cocaine use is a structurally determined socialisation pattern, which we theorise as a ‘top‐boy’ culture. This culture is anchored in status‐seeking elite school milieus characterised by affluence, heavy partying, and exclusive homosocial networks. Boys invested in this culture may engage in cocaine use to signal membership and to mimic the hallmark of ‘ease’, in accordance with a rather orthodox type of masculinity. Whereas youth cultures often represent pockets of resistance to traditional hierarchies, this culture instead seems to strengthen such established hierarchical arrangements.

## 1 Introduction

When you’re on cocaine, you totally feel like a king! You get a lot more confidence. Like, “I’m the most handsome, the coolest, the best”. And you’ve got so much energy. No one can stop you. These feelings—it’s awesome! (Simen)

Cocaine, which stimulates an overproduction of dopamine that typically manifests in a pleasurable elevation in mood and increased levels of energy and self‐esteem (Coomber et al. 2013), is now the second‐most‐used illicit drug in Europe (EMCDDA 2023). Norway as well has seen a strong increase in cocaine use, and in the capital, Oslo, nearly one in four boys in the third year of high school reported using cocaine in a recent study (Bakken 2023).

Youth cocaine use is concerning because of the drug's extreme addictive potential (EMCDDA 2023) and latent health effects such as heart attacks, seizures, and strokes (NIDA 2024). So far, however, we have limited knowledge about which sociocultural components are fuelling youth cocaine consumption.

In this article, we will investigate how cocaine use may be culturally and socially anchored in certain high school cultures. We draw on interview data from a large‐scale qualitative project on youth drug culture; participants who stated that they had used cocaine (25%) made up the focal sample. Most were boys from upper‐middle class and upper‐class backgrounds, and they went to prestigious Oslo schools. We ask: What characterises the youth cultures at these high schools?

### 1.1 Cocaine Use as a Cultural Practice

Our study contributes to the understanding of cocaine use as a cultural practice intertwined with class and gender dynamics. We draw on Bourdieu's concepts of *field*, *habitus* and *capital* (1984, 1990) to situate youth cocaine use and to show how it may reflect and reinforce social hierarchies. According to Bourdieu, all social fields, including high school cultures, are regulated by certain practices and logics, often characterised by actors' struggles to achieve valued goals and positions (Bourdieu and Wacquant 1992). These actors' potential success may reflect the amount of relevant capital they possess (Dumais 2002). We see this ‘field logic’ reflected in studies of school cultures showing widespread agreement among students across the status hierarchy about who are considered the ‘popular ones’ and what confers social status (Armstrong and Hamilton 2013; Milner 2013). This insight infers that youth may also draw on the ‘cultural repertoires’ they have available (Lamont and Molnár 2002) to assign cocaine use with social and symbolic meanings.

Whether investigating increasingly normalised youth cannabis use (Järvinen and Demant 2011; Shildrick 2002) or crack cocaine use among more marginalised groups (Copes 2016), studies show that the use of illegal drugs, while often associated with stigma and negative sanctions, will also be perceived as socially rewarding for users. Engaging in drug use, for instance, may be a powerful symbol of group membership (Collins 2004). While few previous studies have explored cocaine use among youth, studies of adult cocaine users suggest that such usage often occurs in clubbing contexts in the nighttime economy, typically combined with heavy drinking (Edland‐Gryt 2021; Ramo et al. 2011). The stimulating effects paired with the illegality of cocaine reportedly foster feelings of control and reinforce unity in such settings (Darcy 2019; Edland‐Gryt 2021). Whether these findings transfer to youth—who typically have limited access to the nighttime economy—remains to be explored, although research has established that youth are particularly sensitive to peer dynamics of inclusion and exclusion (Laursen and Veenstra 2021; Milner 2013). Thus, youth might engage in drug use to gain social acceptance and signal group belonging.

### 1.2 ‘Doing’ Cocaine, ‘Doing’ Class and Gender

In Norway, youth cocaine use seems to be most prevalent in affluent areas (Pedersen et al. 2025), where the use of illegal drugs typically occurs in sheltered spaces, often in private homes. In contrast, the open drug scenes in Oslo, including the dealing of cannabis and the use of opioids and other drugs, occur in the city's poorer, eastern parts (Sandberg and Pedersen 2011). One reason for this socially skewed pattern of use is that powder cocaine, which dominates in the Nordic countries, is expensive (Neegaard 2023). These wealthy areas are also characterised by heavier drinking and intense partying practices, which often co‐occur with cocaine use (Pedersen et al. 2025). While at odds with the general decline in youth alcohol consumption (Whitaker et al. 2023), this pattern of excess resonates with Luthar's (2003) and Armstrong and Hamilton's (2013) findings that affluent environments are often associated with high levels of youth substance use. This situation may reflect youths' access to economic capital but could also be a response to such environments' often‐intense achievement focus and social pressures, in which drugs may become means of coping and navigating peer group dynamics and social status. As such, cocaine use may be considered a situated class accomplishment (West and Fenstermaker 2007). We have seen related symbolic expressions of affluence in Norwegian studies of economic elite cultures. Flemmen et al. (2018) showed how those who were rich in economic capital more often demonstrated a bodily oriented and expensive quest for excitement. Jarness et al. (2019, 1415) showed how students in such milieus sought a natural style of luxury, which ideally should be displayed on a thin yet athletic body. Such style had to seem ‘authentic’, however, and students ‘trying too hard’ were looked down on. Additionally, Bourdieu's (1984) concept of ‘ease’ informs our analysis, reflecting the often‐challenging process of acquiring a natural sense of belonging in elite institutions. Khan (2011) further theorised ease as a bodily imprinted and socially produced sense of comfort, confidence, and belonging. Ease entails a self‐presentation of unforced effortlessness that in turn influences interactions and provides opportunities in ‘just about any social situation’ (Khan 2011, 15). Because ease often reflects one's socioeconomic status, ease is essentially the ‘practice of privilege’ (Khan 2011, 15). Thus, while ease is socially important, particularly in elite school environments, ease may be anything but easy. Moreover, from Persson's (2021), who recently found ‘confident ease’ to be the hallmark among boys in an economically elite Swedish high school, we take that the concept may also be useful to illustrate how class and gender operate as intersected ‘doings’ in such environments (West and Fenstermaker 2007).

Cocaine use is more common among boys than girls in Norway (Pedersen et al. 2025), and may thus also reflect enactments—or ‘doings’—of gender. Previous research on alcohol and drug use has revealed how boundaries of acceptable behaviour are critically shaped by societal norms of gender performance (Hunt and Antin 2019). Drug use may also be a powerful tool for doing gender (Measham 2002). The ‘doing gender’ concept, introduced by West and Zimmerman (1987), refers to the ongoing process through which people actively engage in behaviours and performances that either reinforce or challenge societal norms and expectations associated with gender. Such a perspective emphasises how gender is constructed and perpetuated through actions and interactions within specific social contexts.

To examine how cocaine use may be an expression of doing gender, our analyses have been informed by Anderson’s (2005, 2010) distinction between orthodox and inclusive forms of heterosexual men's performances of normative masculinity. Much like the traditional business masculinity portrayed by Halvorsen and Ljunggren (2021), *orthodox* masculinity is linked to a focus on strength, competitiveness, and other traditional masculine and heteronormative traits, while *inclusive* masculinity refers to boys engaging in more traditionally feminine behaviours and supporting their female peers' performance of more masculine‐coded roles. Many Western societies have seen a general change towards more inclusive forms of masculinity in recent decades (Anderson 2010). Yet, in Norway, which is widely considered a gender‐equal nation, peer socialisation among high schoolers is still largely organised by gender, and gendered double standards prevail, even in seemingly permissive intoxication and hookup contexts (Fjær et al. 2015; Tokle et al. 2023). In these contexts, so‐called heroic drinking, which implies drinking for the purpose of getting drunk (Demant and Törrönen 2011), is typical, although some research suggests that such gender differences are diminishing (Hunt and Antin 2019).

In the current study, we integrate these understandings to investigate whether patterns of cocaine use may operate as a mean of ‘doing class and gender’. Since contexts are implicated in how gender and class are conducted (West and Fenstermaker 2007), we also explore how youths' particular institutional contexts may shape these ‘doings’.

### 1.3 The High School Context: A Ritualised Elite Graduation Celebration

Because peer socialisation and peer practices are typically defined by the schools that youths attend (Ennett and Bauman 1994), school cultures are strong predictors of youths' alcohol and drug usage (Pedersen et al. 2025). As Flemmen et al. (2018, 145) note, Norway is ‘a comparatively egalitarian society—characterised by, among other things, relatively compressed social inequalities and highly prevalent egalitarian sentiments’. But Oslo is marked by a stark divide between its affluent western and poorer eastern sections (Haandrikman et al. 2023). Oslo's schools reflect this situation, attracting students with varying economic and cultural capital (Jarness et al. 2019; Pedersen et al. 2018). While most schools are public and organised into academic tracks based on lower‐secondary grades, schools in wealthier, central areas are generally seen as more elite due to their location and historical prestige and their students' socioeconomic backgrounds (Halvorsen and Ljunggren 2021; Jarness et al. 2019).

A defining aspect of socialisation throughout high school in Norway is the preparation for the graduation celebration, which ends in a 4‐week period of partying. The Norwegian name for this period is *russetid*, modelled on a university matriculation ritual called *cornua depositurus* (Fjær et al. 2015). The practice is most intense in Oslo's wealthiest areas. Students often form gender‐divided groups by the start of high school, and much of the socialising and partying is organised through such groups. Membership may be associated with much prestige. Participants often buy old buses, which they refurbish to become rolling nightclubs, implying large‐scale economic investments. Like college fraternities (Dekeseredy and Schwartz 1993), the all‐male bus groups are well known for their orthodox performances of masculinity and heavy alcohol consumption (Fjær et al. 2015).

By approaching cocaine use as a cultural practice anchored in certain high school cultures, we make two main contributions. First, we show how the key driver of youth cocaine use is a structurally determined socialisation pattern anchored in status‐seeking elite milieus. Second, we coin the concept of *‘top‐boy’ culture* to capture this milieu. In our empirical analysis, we identify key elements of this youth culture and show how boys invested in this culture may engage in cocaine use to signal membership and a rather orthodox type of masculinity.

## 2 Data and Method

Our analysis is based on a research project on sexual and intoxication cultures in Norway. As part of the project, 127 youth (64 boys), aged 17–19, were interviewed individually about their experiences of cocaine use. Approximately 25% (*N* = 32; 28 boys) stated that they had used cocaine. These participants are the focal sample and will be fleshed out further in the empirical analysis.

The total sample was purposely recruited from 16 high schools in different areas of Oslo and seven schools in smaller cities and rural areas to ensure heterogeneity in socioeconomic status and ethnicity. Initially, we contacted six schools in different areas of Oslo that then granted us permission to present the study for students during classes. We introduced the project to school staff and possible participants as an interview study aimed at understanding the social dynamics of youth sex and drug use. Students were eager to participate; approximately half the sample (and most of the focal sample) was recruited this way. To explore emerging patterns in cocaine use and to safeguard the participants' anonymity, the remaining half were recruited from the other schools through the snowballing and extended‐network approaches.

A trained group of researchers conducted face‐to‐face interviews, most often in private rooms within school premises. We used a semi‐structured guide to explore key themes such as cocaine use. To situate participants within their broader social context, we collected information on their socioeconomic background, schools and peer relations. Throughout, we encouraged them to talk freely and provide examples, but also to refrain from answering questions they deemed uncomfortable. We had a debriefing session to disclose any potential distress; participants who indicated a need for support were encouraged to seek assistance. Interviews lasted between one and 2 hours and were audiotaped and transcribed verbatim.

The study was evaluated and recommended by SIKT (the Norwegian Agency for Shared Services in Education and Research; ref. 200363) and was conducted in accordance with the ethical guidelines of the National Committee for Research Ethics in the Social Sciences and Humanities. Participants provided informed and active consent for participation based on detailed oral and written information about the study design. Information on substance use and mental health support services was provided in the information sheets. To ensure the participants' anonymity, we have used pseudonyms in this paper and have omitted school names and identifying details.

Transcripts were coded in NVivo, then analysed in a three‐step process. First, we read and mapped all coded transcripts to categorise the non‐users’ accounts of others' cocaine use. Secondly, to gain a more robust understanding of the cultural context of such use, we engaged more in depth with the transcripts and field notes from the focal sample who had stated that they had used cocaine. Accounts were sorted into the following themes: social class, social status, school environment, socialisation practices, intoxication practices, cocaine practices and cocaine's broader meanings. Themes were reviewed and empirical tendencies highlighted. Finally, the context and the most important cocaine‐related excerpts were linked and developed into four themes that we understand as key characteristics associated with cocaine use.

We will start by unpacking these themes before further conceptualising what we consider a ‘top‐boy’ culture. All quotations reflect core empirical tendencies. Extracts have been translated from Norwegian to English by the authors and lightly edited for clarity.

## 3 A Top‐Boy Culture Driving Cocaine Use

Three‐quarters of the total sample stated that they had never used cocaine, yet they demonstrated a shared understanding of the ‘youth cocaine phenomenon’ as linked to *certain* youths in *certain* social setups. Boyhood, money, and excessive party rituals were recurring themes when participants depicted the typical cocaine user. While the non‐users’ narratives may be interpreted as boundary work and othering, the focal sample who stated having used cocaine came through as a relatively homogenous sample, habitus‐wise. Most were boys (28 of 32) from upper‐middle or upper‐class (24 of 32) backgrounds who belonged to the white ethnic majority (31 of 32). They also attended a handful of Oslo schools in affluent districts with a sociocultural setup we call ‘top‐boy’ culture, marked by (i) *affluence*; (ii) a *party‐centred culture* with heavy alcohol usage; (iii) *exclusive peer networks*, linked to graduation‐celebration groups; and (iv) *orthodox masculinity norms.* Below we show the interplay between cocaine use and investment in this top‐boy culture.

### 3.1 Affluence

Janne was one of the few girls who had tried cocaine and was involved in the status‐seeking cocaine culture at these schools, which was dominated by boys with both prestige and status. She had an insider perspective, yet talked about the boys in this culture rather ironically:

These boys have watched *Exit* and *Wolf of Wall Street.* They see that [these characters] are successful and have a lot of money and use a lot of cocaine and think, “Awesome! We’ll do that, too!”

Several described the ‘boyish’ cocaine practice as influenced by the cult film *The Wolf of Wall Street* and its semi‐fictional Norwegian equivalent, the *Exit* series. Both examples are depictions of cultures rooted in the upper economic echelons, where ‘greed is good’ and material wealth is the primary indicator of success and power (Friedman and Reeves 2020). The male characters display dominant and risk‐taking behaviour while engaging in semi‐criminal business affairs, extravagant parties, and cocaine use. Money fuels this lifestyle.

The fact that economic capital affected the youths' cocaine consumption is not surprising. Powder cocaine is expensive (Neegaard 2023), and having enough money makes cocaine more accessible. Most of the participants who stated that they had used cocaine came from Oslo's affluent areas. Many had parents with high‐income jobs, lived in large houses, and enjoyed access to expensive vacation houses. While some also hailed from backgrounds rich in cultural capital, this cocaine‐using subculture was largely rooted in schools with students from the middle and upper economic classes. Subsequently, many expressed awareness of their economically privileged positions through statements such as ‘We are well‐to‐do, living well off’ (Elias) or ‘We have plenty of money, a nice house, ample space’ (Martin). Some were more outspoken and upfront about their advantages, such as Nils, who was what might be categorised as an ‘excessive’ cocaine user:

I live in Norway, in Oslo, which is brilliantly cool. I live on the west side, which is even better. I come from a privileged family. I have lots of friends. I do well in school. I’m good looking. So yes, as everything suggests, I’m doing very well.

While such a presentation of self could be considered boastful, these boys typically discussed their backgrounds in a matter‐of‐fact manner, with ease and little affect.

Their typical patterns of poly‐substance use added to the expense. Albert said, ‘You rarely do coke without drinking, and you rarely drink without doing coke; the two come as a pair. [Cocaine] enhances the alcohol experience. You simply feel more intoxicated’. Echoing this quest for excess, Anton described how, during the graduation celebrations, he ‘would use around 1 g of cocaine a night, in addition to much alcohol’. Heavy alcohol consumption and repeated cocaine intake throughout the night make cocaine use a costly habit. Eirik, from western Oslo, said:

Cocaine is common here. Many people spend many, many thousands [of kroner] on it, particularly during the graduation period. The dealers even drive to your house and deliver. So access, I guess, is only difficult for those who don’t have the finances.

Having high social status increased youths' access to cocaine. Albert was extroverted and the leader of a prestigious group at his school. He said, as a reflection of his position, that ‘I've done cocaine around 20 times, but never paid for it’. Gustav, who went to a similar Oslo school, further illuminated how social status could affect one's accessibility to drugs: ‘I noticed it during the graduation period, that other graduation buses wanted to offer us a lot of cocaine, because they wanted to be friends with us’.

While Albert and Gustav, like most of the cocaine users, came from affluent backgrounds, we also found examples where social capital, based on popularity, a broad social network and high ‘bodily capital’ (Mears 2015; Milner 2013), compensated for a lower economic status. Oliver, a popular, good‐looking boy from a more modest middle‐class background than the others, told us that ‘I'll always be offered a line by those who have the money to do whatever they want’. Hence, while economic privileges were typical and were often interlinked with high social status, an affluent background was not a sine qua non for using cocaine.

### 3.2 Party‐Centred Culture

While the broader context of cocaine use was affluence, other facilitators included hard partying and permissive intoxication norms. Martin said:

Everyone from my group parties a lot, typically twice a week, on Friday and Saturday. Every weekend, really. You do what the other guys do. Everyone’s drinking, so you drink. If you don’t drink, you stay at home.

Social interactions outside school hours largely revolved around party settings and rituals of intoxication. Lavrans said, ‘Intoxication is an important part of the social scene. Maybe it’s a bit too important, when you think about how rarely guys [like us] hang out sober outside school. It's rare, very rare’.

The ‘field logic’ was the same across these schools and appeared as follows: Intoxication was a necessity to be included at parties, and being at these parties was a necessity to be included in the popular social groups at school. Institutionalised norms of excessive partying and alcohol consumption in the ‘heroic drinking’ pattern (Demant and Törrönen 2011) seemed to foster cultural acceptance of the recreational use of cocaine as well. Upon entering the institutionalised ‘party pathway’ (Armstrong and Hamilton 2013), many of the boys noted how they had soon become accustomed to the culture's permissive intoxication norms. Eivind said:

When I started high school, I’d only tried cannabis. I always thought I’d stay away from other drugs. But after hearing about the others' experiences with coke, I wanted to try it myself—you know, just to see what it did to me. And since it’s fun, it became normal. Now, I have no idea how many times I’ve done it, but it’s a lot.

While most Norwegian adolescents do not use cocaine, the boys in our focal sample referred to it as something that had become normalised in their institutional context, typically through statements such as the following: ‘Cocaine has become so common. It's something everyone does. Whereas 2 years ago, for me, it was like using heroin’ (August); ‘Everyone uses it, everyone who parties at least’ (Mikkel); and ‘In our group, there are probably only two out of 28 people who've never tried cocaine’ (Thomas). These accounts suggest a pattern of ‘differentiated normalisation’ (Shildrick 2002). The use of cocaine had become normalised within their specific culture.

The narratives of normalisation also became self‐reinforcing by paving the way for novel users and subsequently more cocaine use. Lars illustrated this dynamic:

When you’re in this environment over time, it’s impossible not to have done [cocaine]. Because everyone does it. And when everyone does it, it just seems a lot less dangerous, and there are basically no [social] consequences for doing it, since it’s so normalised.

In this party‐centred culture, participants portrayed cocaine as an ideal drug to demonstrate social mastery. The substance produces an intense euphoric high, combined with more energy, alertness, and confidence (Coomber et al. 2013). These characteristics are highly valued in top‐boy culture. Doing some lines of cocaine provided the participants with energy to keep the party going and to continue drinking throughout the night. As Christian explained:

Without it, you start to struggle around three [a.m.]. It’s kind of an energy supplement, to keep [you] going…especially towards the morning. During the graduation celebration, we had all these hoodies made for us with an inside zippered pocket for cocaine…. When you needed energy, you just did a key [of cocaine].

Alongside the bravura in the boys' reflections on their intoxication practices, they also showed traits of justification and ambiguity:

I guess we *are* more extreme with the amount of alcohol and cocaine [we consume], but we feel it’s so normal, and I don’t feel like it affects our health. But when I’ve gone out with others from different schools, and I bring ten beers, and they bring four…I can see that the party culture is calmer in other areas (Hugo).

Accounts such as Hugo's displayed awareness of their substance consumption being on the more excessive and potentially riskier end of the continuum. These findings also underscore how peer influences are paramount during adolescence (Laursen and Veenstra 2021).

### 3.3 Top‐Level Networks

Another top‐boy culture characteristic that drove cocaine use was the existence of exclusive social networks across these schools. The backdrop was the ritualised *russetid* traditions, which have a long history of prestige in Norway. Elias said:

We have these strong traditions and cultures in our school, and these *russe* buses. It’s a clique system. You easily see who’s on the inside and who’s on the outside. We’re on top, so for me the social hierarchy is nothing bad, but I guess if you asked someone who doesn’t have the social circle I do, they’d say it’s terrifying, and I get that.

As Elias described, peer socialisation among youth invested in this culture was heavily scripted in accordance with these schools' established traditions and rituals. Closed social groups were established early on, based on gender and social status (Milner 2013), often with the aim of buying a bus in the final year. While financial comfort was the unspoken requirement for membership, high ‘bodily capital’ was also valuable (Mears 2015). Sebastian said, ‘Its “pretty privilege”; obviously, it's a well‐known fact that it’s easier to get into groups if you're good looking’. Underscored by alphabetical order, group membership was an indicator of social status, as Nils described:

We’re [a group of] 28 boys from Oslo’s west side, and we’re at the top; we’re the A group. There’s also a similar group of [top] girls. Then you have the B and C groups.… It’s just ridiculous how strong this hierarchy is.

These customised buses can hold a maximum of 28 passengers, limiting the number of members in a bus group. The hierarchical structure and the restricted number of participants created exclusivity and increased the significance of belonging. Simen, one of the top boys who used cocaine, said, ‘Once the A, B, C, D order is set, it's set in stone. After that, there's no chance for mobility’.

For boys across these schools, inclusion in an all‐male group was a matter of being socialised into a tight friendship group, gaining access to attractive parties, and pursuing status positioning. Another of the cocaine‐using boys, Martin, alluded to this situation when he said, ‘I'm quite popular. I'm on the A bus, and I have lots of friends. I always have a busy schedule on weekends’.

This broader social setup—the visible status hierarchies and exclusive peer groups, as well as the pressure such socialisation produced—fuelled cocaine use. Such usage was a way for boys to engage in socially valuable bonding. Ola said:

I’ve become good friends with all the guys I’ve done cocaine with. It’s like a brotherhood. We share something special; it creates unity. That’s what I love about it: the ritual of buying some grams and doing lines with the guys. It’s great.

Samuel shared this view: ‘Doing cocaine is about being together. It's special. You share something’. These excerpts reflect how substance use rituals can mark boundaries of inclusion (Collins 2004).

The social dimension of belonging and the reinforced unity with ‘the guys’ were recurring themes when the boys discussed cocaine's appeal. Notably, cocaine's illegal status only added to this appeal. Stian said, ‘It's exciting to do things that are illegal’. As Katz (2013) has theorised, engaging in illegal acts may be seductive because of the excitement involved. In our study, engaging in the cocaine ritual together also signified mutual trust due to the risk associated with illegal acts. Anton said:

What we usually do is, we announce at the pre‐party, like, “Who wants some?” Then we message the dealer, like, “We want 10 g”. When the guys who’ve bought [cocaine] together team up during the night to do it, it creates a little camaraderie.

The mechanisms that made buying and using cocaine an inclusion ritual for users also made it a ritual of exclusion. Boys who aspired to gain access to a group could consider the social risk of not engaging as overshadowing the potential health and other risks of cocaine use. The aim of belonging and the fear of exclusion thus may be overlapping drivers for cocaine use in top‐boy culture. Karim's story added to this argument:

I really tried to fit in, to socialise. I was so desperate that I tried cocaine. But the second time, I blacked out completely. It turns out I made a move on a girl that one of the guys had been talking to, and that was it.… The little residue of hope I had of being considered one of them was gone. I changed schools soon after and haven’t touched cocaine since.

Karim's case illuminates how cocaine use may be adopted as a means of fitting in or gaining social status in exclusive homosocial networks. Notably, his story also indicates that cocaine use per se is not enough to gain entrance. While Karim, like the others, came from an affluent background, he was less well connected. Karim was also the only participant from a minority background who stated that he had used cocaine, which reflected how the top‐boy culture is largely dominated by white‐majority boys. While his ethnicity may have contributed to his outsider position (Milner 2013), ‘trying too hard’ (Jarness et al. 2019, 1415) and hitting on a girl ‘claimed’ by a boy higher up in the hierarchy also gravely conflicted with other codes of conduct in top‐boy culture, namely that of ‘ease’ and the brotherhood support system. Breaking these norms excluded Karim from the top‐boy culture at his school.

### 3.4 Masculinity

In addition to affluence, permissive intoxication practises, and exclusive networks, orthodox masculinity norms were an important marker of cocaine use in top‐boy culture.

Girls who attended these schools with abundant cocaine usage described the practice through statements such as ‘Cocaine is normal; it's everywhere at parties’ (Marte) and ‘I know plenty [of people] who do it; it's extremely common’ (Lea). The narrative of normalisation changed when gender was added to the equation, however: ‘It's mainly boys who do [cocaine]’ (Marte) and ‘It's common among boys, not the girls’ (Lea).

This gender‐differentiated portrayal of cocaine use, with the practise being primarily reserved for boys, was less evident with alcohol use in these Oslo schools (Pedersen et al. 2025). Yet, while the drinking norms were permissive across gender (Hunt and Antin 2019), boys were subjected to more peer pressure to engage in combined heavy drinking and cocaine use. Monica, who was in a popular all‐female celebration group, said:

There’s more pressure to drink and do drugs among the boys than the girls. No one thinks a girl is boring if she’s sober when she goes out. But I can’t say the same for the boys.

Participants in Oslo schools with top‐boy culture noted how their male peers more often experienced pressure to use cocaine, and of being ‘in the know’, to use Thornton's (1997) phrase. The typical effects of cocaine aligned with the norms of masculinity in elite institutions, such as extroversion and navigating social interactions with utter confidence—what we call ‘ease’ (Khan 2011). Several described how boys with high social status were characterised by ease. Thomas said:

On weekends, being invited to several parties indicates status. Those at the top are the most outgoing, talk to the most people, and have the largest social circle. They automatically take control in social settings.

While acting with ease opened chances for social status and influence in social settings, ease was also an embodied reflection of the boys' socioeconomic status (Khan 2011). Ludvik, one of the most popular boys at his school, said:

In my group, approximately half the boys who do coke, do it because they struggle with self‐confidence. Coke makes it easier for them to talk to girls. For me, self‐confidence has never been an issue. Like, many say that when they’re on coke, they *feel* like the coolest person in the room. As for me, I *know* I’m the coolest person in the room.

While some of the top‐tier boys, like Ludvik, who was rich in both economic and social capital, described how they already had ‘what it took’ to demonstrate confident ease (Khan and Jerolmack 2013), more socially insecure boys could use cocaine to *mimic* ease. Erik said, ‘I'm not so nervous when I drink and do coke. Plus, when you're on cocaine, your self‐confidence goes up’. Ola said, ‘I become a different person when I'm on coke…and that's what I like about it’. Doing a line of cocaine was an easy way for boys to overcome their social insecurity and boost their self‐confidence. Hugo said:

What’s important is to be a positive person—one who creates life, dares to take up space, dares to speak up—and with cocaine you get such sick energy. You just keep going, and it feels like you can master anything.

The use of cocaine to display mastery of ‘confident ease’ (Persson 2021) was particularly useful in mixed‐gender party settings. Since peer groups and pre‐parties typically were gender segregated, gender‐mixed parties were the youths' primary ‘hookup’ arena. Gendered expectations in these hookup settings contributed to the boys' cocaine use. Elias said:

The desire for coke arises particularly when girls are present, which I think is indicative of the need to boost your confidence. The responsibility for making it a good party rests on us guys. Girls see it as our obligation. They expect us to be funny and cool.

The use of cocaine to *mimic* ease around girls at parties was often about being able to hook up and have sex. In this heterosexual and status‐driven context, double standards prevailed, and boys were generally considered the initiators of sex (Armstrong and Hamilton 2013). Reflecting Mears' (2015) description of elite males' appropriation of girls' bodily capital, for boys, having a high ‘body count’ signified social status, especially if the sexual interactions had involved girls considered pretty and of high status. To align with these rather orthodox masculinity norms (Anderson 2005), boys could use cocaine to ‘man up’ in their sexual confidence. Kristian said, ‘I felt such an effect from using it. I felt like the king. And when you feel more superior, you have a greater chance of hooking up with the girls you want’. Additionally, girls invested in this culture noted that this strategy could make boys overly self‐confident. Kaia said, ‘On coke, they're pushier and don't quite take no for an answer’. For boys with lower social status, we found examples of how being ‘too pushy’ while on cocaine was discouraged by exclusion and the stigma of being labelled a ‘creep’. Reflecting the strong codes of brotherhood, however, popular boys typically had their friends' unconditional backing. This male peer‐support system, which made high‐status boys more untouchable (Dekeseredy and Schwartz 1993), also illustrates how the top‐boy culture serves to maintain and reproduce social inequalities within these status‐driven schools (Armstrong and Hamilton 2013).

## 4 Discussion

In this study, we have shown how cocaine use is culturally and socially anchored in the graduation celebration at a handful of Oslo schools catering to the upper economic classes. Our analysis has illustrated, step by step, how the co‐occurrence of affluence, exclusive hierarchical networks, excessive party practices, and orthodox masculinity norms may reinforce cocaine use. Put simply, we found that wealth provided access to cocaine, and, by virtue of its effects, cocaine use gave youth, mostly boys, a tool to navigate according to the peer, class and gender logics of the social setup, which we theorise as a top‐boy culture. In the following, we will expand on the implications and key contributions of our findings.

### 4.1 Theorising Top‐Boy Culture

A primary contribution is our coining of the concept of top‐boy culture, which refers to hierarchical school environments endowed with considerable economic capital, much prestige, and exclusive all‐male peer‐group arrangements in which social status and masculinity play key roles for belonging. To demonstrate the necessary commitment, boys invested in this culture engage in ‘heroic’ partying and accept codes of brotherhood and male peer support linked to orthodox masculinity (Anderson 2005, 2010). This status‐seeking culture is based on a structural setup at these schools that influences the social experiences of all students but benefits only affluent and well‐connected youth (see also Armstrong and Hamilton 2013). We believe the concept has relevance when analysing social‐status dynamics within similar networks, such as private clubs, secret societies (Scott 2011), college fraternities (Dekeseredy and Schwartz 1993), and sororities (Armstrong and Hamilton 2013).

### 4.2 Cocaine Use as a Cultural Practise in Top‐Boy Culture

By approaching cocaine use as a cultural practise, our study has contributed important knowledge about which sociocultural components fuel the significantly higher rates of cocaine use among boys in affluent Oslo schools (Pedersen et al. 2025). Reflecting Bourdieu’s (1984, 1990) notion of how class, power, and status dynamics shape people's modes of conduct, our findings suggest that top‐boy culture is a key driver of cocaine use. Boys who reported using cocaine seemingly engaged in behaviours that aligned with key norms in their social milieu (Lamont and Molnár 2002). Reflecting other elite youth contexts (Armstrong and Hamilton 2013), parties are important for socialisation and status attainment in top‐boy culture. Playing by the ‘rules of the field’ involved excessive partying, based on permissive norms. Cocaine's effects made it easier for boys to regulate their level of alcohol intoxication and to boost their energy to ‘keep the party going’ (Darcy 2019). Notions of cocaine use as ‘normalised’ (Shildrick 2002) served as justification, but subsequently also paved the way for more social pressures to use the drug to be considered ‘in the know’ (Thornton 1997).

The importance of social status and the wish to be part of exclusive peer networks formed a vital backdrop for each of these factors. Cocaine use was a way to reinforce bonding and unity (Collins 2004; Edland‐Gryt 2021), while not using cocaine might signal poor group investment and lead to exclusion in top‐boy culture. Our findings thus align with previous research that has described affluent environments as status‐seeking (Jarness et al. 2019) and high‐risk for youth substance use (Armstrong and Hamilton 2013; Luthar 2003).

### 4.3 Cocaine Use as ‘Doing’ Top‐Boy Culture

Another key contribution of this study is to show how youth cocaine use in top‐boy culture is deeply entwined with the performance of class and gender (West and Fenstermaker 2007), and ultimately how these performances serve to maintain and reproduce social inequalities.

Reflecting our title's metaphorical reference to *The Wolf of Wall Street*, an important factor was money as a marker of success. Much like Veblen's (1899) observation that some goods become desirable because they are costly and therefore act as an advertisement of the possessor's wealth, managing the high cost of cocaine was a symbolic expression of economic capital. The visible and widespread use of cocaine in top‐boy culture may thus be considered at odds with broader elite cultures' changes towards more subtle expressions of economic capital (Friedman and Reeves 2020).

While the upper tiers typically came from backgrounds rich in economic capital in our study, we also found exceptions of boys from more modest middle‐class backgrounds with high social status. Despite having less money than their peers, they also described having easy access to cocaine. These examples illustrate (1) that the school culture's large number of affluent members increased the overall accessibility of cocaine and (2) how different capital forms intersected (Bourdieu 1984). Similarly to findings from other status‐seeking school contexts, social status, or popularity, was intrinsically linked to having high social capital attained through a large social network (Milner 2013), mastering the right physical appearance (Jarness et al. 2019), and hooking up with the ‘right’ girls (Mears 2015). These expressions of bodily capital in top‐boy culture may also resonate with Khan's (2011) and Persson's (2021) views of ease as a display of superiority in hierarchical interactions.

‘Ease’ plays a central role in the gendered self‐presentation and social status of top‐boy culture. Our findings thus underscore Persson's (2021) point that ease is shaped both by embodied dispositions and the specific context. This study, however, extends previous research by linking ease to cocaine use. Our findings suggest that while cocaine use among many top‐tier boys could be interpreted as an expression of ease ‘no matter the context’ (Khan 2011, 15), more socially insecure boys used cocaine to *mimic* ease to conform to the top‐boy culture's orthodox masculinity norms (Anderson 2005): Cocaine use allowed boys to sustain heavy drinking, to reinforce participation in homosocial networks, and to gain the necessary confidence for initiating hookups per the gendered expectations ascribed to boys in top‐boy culture. Put differently, cocaine use may serve as a symbolic performance of orthodox masculinities by embodying traits such as risk‐taking, social status, and dominance.

Interestingly, the ‘homosocial vibe’ (Gottzén et al. 2019) and the orthodox masculinity norms we have identified in top‐boy culture starkly contrast with the established Nordic progressive ideal of a ‘softer’ and more inclusive masculinity. Previous studies have linked cocaine use to club cultures (Edland‐Gryt 2021). Thornton (1997) described how such club cultures could form pockets of resistance in a society with stark class differences. In contrast, our study has revealed how cocaine usage in top‐boy culture seamlessly consolidates class differences through the culture's hierarchical arrangements. Yet, the heterosexual and orthodox masculinities that are forged through all‐male group socialisation in top‐boy culture may indeed be seen as pockets of resistance, but as resistance articulated against ‘politically correct’ norms of gender equality, as well as against the more inclusive forms of masculinity that have developed over the past few decades (Anderson 2010).

The broader cultural and political backdrop of our findings may thus be the resurgence of traditional and often hypermasculine ideals characterised by dominance and self‐reliance, as popularised by influencers such as Andrew Tate and the recent right‐wing mobilisation among young males in many countries, including Norway (Kleven 2024). The recent Norwegian elections featured a highly gender‐polarised political landscape in which few males voted for parties on the ‘red‐green axis’, which have promoted gender equality and a more inclusive masculinity expression (Hesstvedt et al. 2023). These broader currents may suggest that cocaine use's symbolic appeal can extend beyond boys invested in top‐boy culture at prestigious Oslo schools.

In conclusion, our findings indicate an urgent need for targeted prevention strategies aimed at youths in school milieus associated with affluence, excessive party practices and exclusive school rituals.

## Ethics Statement

This research was produced from a study that received ethical approval from SIKT (the Norwegian Agency for Shared Services in Education and Research; ref. 200363) and has been conducted in accordance with the ethical guidelines from the National Committee for Research Ethics in the Social Sciences and Humanities.

## Conflicts of Interest

The authors declare no conflicts of interest.

## Data Availability

Research data are not shared.
